# CD1d affects the proliferation, migration, and apoptosis of human papillary thyroid carcinoma TPC-1 cells via regulating MAPK/NF-κB signaling pathway

**DOI:** 10.1515/med-2024-0949

**Published:** 2024-06-10

**Authors:** Qingyuan Liu, Tong Zhai, Lei Ma, Qilun Liu

**Affiliations:** The Third Departments of Tumor Surgery, General Hospital of Ningxia Medical University, Yinchuan City, Ningxia, China; Department of Breast, Baoji Central Hospital, Baoji City, Shanxi, China

**Keywords:** CD1d, papillary thyroid carcinoma, TPC-1 cells, MAPK/NF-κB signaling pathway, RNA interference

## Abstract

The study aimed to investigate the effect of CD1d down-regulation on the proliferation, migration, and apoptosis of papillary thyroid carcinoma cells and explore the underlying mechanism. CD1d expression was silenced in TPC-1 cells by transfection of CD1d siRNA lentivirus. The proliferation, apoptosis rate, and migration ability of TPC-1 cells were detected by CCK-8 assay, flow cytometry, and scratch assay, respectively. Western blot and qPCR analyses were performed to detect the expression of related proteins. CD1d was highly expressed in TPC-1 cells. Down-regulation of CD1d significantly decreased ALMS1, CDKN3, CDK6, Ki-67, Bcl2 expression, increased Bax and Caspase 3 expression (all *P* < 0.05), and decreased the migration ability of TPC-1 cells. Gene ontology and Kyoto Encyclopedia of Genes and Genomes (KEGG) analysis were performed to identify the relevant signaling pathways. KEGG pathway enrichment analysis showed that the differentially expressed genes were mainly enriched in MAPK and NF-κB signaling pathways. Our findings suggest that CD1d down-regulation inhibited the proliferation and migration abilities of TPC-1 cells, increased cell apoptosis possibly via the MAPK/NF-κB signaling pathway.

## 1 Introduction

In the last few decades, thyroid cancer is becoming more prevalent, which is gradually becoming the most common malignant tumor of the head and neck regions, and also the most common endocrine system cancer. The incidence of thyroid cancer has shown an increasing trend since the end of the last century, and patients with thyroid cancer tend to be younger [1–3]. Thyroid cancer arises from the follicular epithelial cells or parafollicular cells [4]. It is estimated that the incidence of thyroid cancer has increased dramatically in recent decades [3,5–8]. The overall prognosis of thyroid cancer is different, with both poor and good outcomes observed, and the reasons for this are still unclear.

With the advancement of science and technology, exploration of the molecular mechanisms underlying the development of thyroid cancer is ongoing. Several tumor suppressor genes and oncogenes have been confirmed and reported, including BRAF, P53, RAS, BCL-2, TSH, matrix metalloproteinases, Fas/Fas ligand, and some relatively newly discovered genes, such as MET, GSP, TRK, and RB [9–11].

CD1 genes are present in humans and mice. The human genome contains five CD1 family genes organized in a cluster on chromosome 1. CD1d exerts its role by binding exogenous glycolipid ligand α-galactose ceramide and endogenous ligand sphingolipid isogloboside b3, thus inducing the production of cytokines. CD1d can act both directly and indirectly to human body, and is involved in multiple molecular and physiological activities [12,13]. Previous studies on CD1d mainly focused on the role of CD1d on various inflammatory disorders, but the specific mechanisms have not been studied in depth. A few experiments have verified the relationships between CD1d and tumors; however, relationships between CD1d and papillary thyroid carcinoma (PTC) have not yet been reported. The biological role of CD1d is controversial, and its role in different tumors is not the same. It has been reported that CD1d has significance in the occurrence and development of various tumors, and plays an important regulatory role. The expression and clinical significance of CD1D in human papillary thyroid carcinoma (PTC) are still unclear, and its role, molecular mechanism and biological function in PTC are still worth exploring.

Therefore, this study aimed to analyze the effect of CD1d on the progression of PTC cells, and explore the potential underlying mechanism, thus providing a potential molecular target.

## 2 Materials and methods

### 2.1 Materials

Cell lines used in this study included human PTC cell line (TPC-1; No. BNCC 337912, Beina Chuanglian Biotechnology Co, Ltd, Beijing, China) and human thyroid follicular epithelial cell line (Nthy-ori 3-1; No. BNCC 337912, Beina Chuanglian Biotechnology Co, Ltd, Beijing, China). Reagents and instruments used in this study were as follows: small interfering RNA targeting CD1d (CD1d-siRNA, Shanghai Gene Pharma Co., Ltd, Shanghai, China), the negative control (NC)-siRNA (Shanghai Gene Pharma Co., Ltd, Shanghai, China), Cell Counting Kit-8 (CCK8, C0038, Beyotime Biotechnology, Shanghai, China), horseradish peroxidase-labeled goat anti-rabbit IgG (ab205718, Cambridge, UK), reverse transcription and real-time PCR (RT-PCR) kits (P612-01, Vazyme Biotech Co., Ltd, Nanjing, China), spectrometer (Multiskan FC, Thermo Fisher, UK), RT-PCR instrument (7500fast, Applied Biosystems, CA, USA), fluorescence microscope (DM2700P, Leica, Germany), and flow cytometer (Accuri C6, BD, USA).

### 2.2 Cell culture

TPC-1 cells were cultured in RPMI-1640 medium containing 10% fetal bovine serum, and Nthy-ori-3-1 cells were cultured in dulbecco’s modified eagle medium medium containing 10% fetal bovine serum at 37°C in a 5% CO_2_ incubator. The medium was changed every 2 days, cells were digested and passaged with 0.25% trypsin.

### 2.3 Cell transfection and grouping

Cells in the logarithmic growth phase were taken, digested, and then inoculated on six-well plates overnight. When the cell density was about 50–60%, lentiviral transfection solution containing CD1d-siRNA or NC-siRNA (at a multiplicity of infection of 1:20) was added into the culture dish. After incubation for 8 h, the culture medium was replaced with the complete culture medium. After transfection, culture was continued for 48 h, cells were then collected for subsequent experiments.

### 2.4 Immunocytochemistry

Cells were fixed with 4% paraformaldehyde at room temperature for 20 min, permeabilized in 0.3% Triton X-100 (Solarbio) for 10 min and endogenous peroxidase activity was quenched in 1% H_2_O_2_ for 15 min. Then blocked with 10% normal goat serum (AR0009, Boster, Wuhan, China) for 30 min at 37°C. The cells were then incubated overnight at 4°C with anti-CD1d (66257-1-Ig, ProteinTech Group, Chicago, USA) at a 1/100 dilution. The second antibody (PV-9002, ZSBIO, Beijing, China) was incubated at 37°C for 1 h and then stained with 3, 3′-diaminobenzidine. Nucleus was labeled with hematoxylin. Images were taken with microscope (DM2700P, Leica, Germany).

### 2.5 RNA extraction and qPCR

The collected cells were mixed thoroughly with 700 μL of TRIzol reagent, and total RNA was extracted with chloroform. The RNA concentration and purity were determined using a spectrometer. Synthesis of the cDNA first strand was conducted using the reverse transcription kit according to the manufacturer’s instructions. The reaction solution was prepared with 2 μL 5× gDNA Eraser buffer, 1 μL DNA Eraser, 6 μL Rnase free dH_2_O, and 1 μL total RNA. The reactions were incubated at 37℃ for 12 min, followed by 85℃ for 5 s, and 4℃ to terminate the reaction. The cDNA was then used as the template for the qPCR reaction. The reaction conditions were as follows: 45℃ for 3 min, followed by 40 cycles of 95℃ for 2 min, 95℃ for 15 s, 65℃ for 2 min. The sequences of the primers used for PCR were as follows: CD1d: forward: 5′-GTATCTCCGAGCAACCCTGGAT-3 and reverse: 5′-AGGACTGCCAAGGCAATCAAGC-3′; β-actin: forward: 5′-CACGAAACTCCTTCAACTCC-3′ and reverse: 5′-CATACTCCTGCTTGCTGATC-3′. The relative quantification of gene expression was calculated using the 2^−ΔΔCT^ method.

### 2.6 Detection of p-p38 MAPK, p38 MAPK, HRAS, p-NF-κB p65, NF-κB p65 expression using western blot analysis

After 48 h of transfection, cells were collected, placed in 0.4 mL RIPA lysis buffer, and lysed on ice for 30 min. After centrifugation at 12,000 rpm for 18 min at 4°C, the supernatant was collected, and quantified with BCA protein. Then the supernatant was mixed with 5× sample buffer, boiled at 100°C for 8 min, and stored at −20°C. The samples were subjected to sodium dodecyl sulfate-polyacrylamide gel electrophoresis gel electrophoresis, and electro-transferred to polyvinylidene fluoride membrane, which was then blocked with 5% skimmed milk for 1 h. After washing three times with phosphate buffered solution with tween (PBST) (5 min each time), the membranes were incubated with primary antibodies against p-p38 MAPK, p38 MAPK, HRAS, p-NF-κB p65, NF-κB p65, β-actin overnight at 4°C. After washing three times with PBST (5 min each time), the membranes were incubated with secondary antibody (1:5,000) at room temperature for 2 h, followed by washing three times with PBST for 10 min each time. The protein bands were visualized by an enhanced chemiluminescence. The gray values of the protein bands were analyzed using the Image-pro plus software, and the relative expression levels of proteins in each group were represented by the ratio of the gray value of the target protein and the β-actin

### 2.7 Determination of cell viability by CCK-8 method

Cells in each group were washed twice with pre-chilled PBS, digested with trypsin and centrifuged, the supernatant was then discarded. Cell suspensions were seeded into a 96-well plate at 1 × 10^4^ cells per well and incubated for 12 h. About 100 μL of cell suspensions were added to each well of the 96-well plate, and three replicates were taken for each group. At 24, 48, and 96 h, the optical density (OD) values at 450 nm were measured, and the data were recorded.

### 2.8 Detection of cell migration and invasion by scratch assay

Cells in each group were inoculated in a six-well plate. After cells exhibited adherent growth and spread over the well bottom, uniform horizontal lines at a distance of 1 cm were made on the back of the plate with a marker, after that, the 96-well plate was placed in an incubator for further culture. Photographs were taken at 0, 24, and 48 h after scratching. The experiment was performed in triplicate.

### 2.9 Detection of cell apoptosis by flow cytometry

The apoptotic of cells were detected using annexin V-FITC and propidium iodide (PI) double staining [14]. Cells in each group were inoculated in a six-well plate. After 24 h of growth, cells were adherent to the plate wall, culture was continued for 48 h. Cells in each group were collected, washed three times with PBS, and centrifuged at 1,200 rpm at 4℃ for 5 min, the precipitates were then taken. Annexin V-FITC and PI staining were performed according to kit instructions. After washing three times with PBS, cell pellets were resuspended in 100 μL of labeling solution, incubated for 30 min at 4℃ in the dark, centrifuged at 1,200 rpm for 5 min, and then rinsed gently three times with buffer. Cells were analyzed by flow cytometry. In flow cytometry plots, cells were divided into four quadrants. The lower right quadrant represents early apoptosis (Annexin-FITC +/PI−), the upper right quadrant represents late apoptotic cells (Annexin V-FITC+/PI+), the lower left quadrant represents normal cells (Annexin V-FITC-/PI-), and the upper left quadrant represents necrotic cells (Annexin V-FITC-/PI+). Apoptosis rate was calculated as summation of early and late apoptotic cells.

### 2.10 Bioinformatics analysis

Transcriptome sequencing (RNA-Seq) was used to screen out differentially expressed genes (DEGs) between the CD1d-siRNA group and NC-siRNA group. Gene ontology (GO) enrichment analysis of the DEGs was implemented by the top GO package. Kyoto Encyclopedia of Genes and Genomes (KEGG) enrichment analysis was performed using the KOBAS software.

### 2.11 Statistical analysis

Statistical analyses were performed using SPSS 19.0 statistics software. Data are expressed as mean ± SD. The *t*-test was performed for the comparison between two groups. One-way analysis of variance was used for multiple comparisons. Three replicates were performed for each experiment. A value of *P* < 0.05 indicated statistical significance.

## 3 Results

### 3.1 Increased CD1d expression in TPC-1 cells

**Figure 1:**
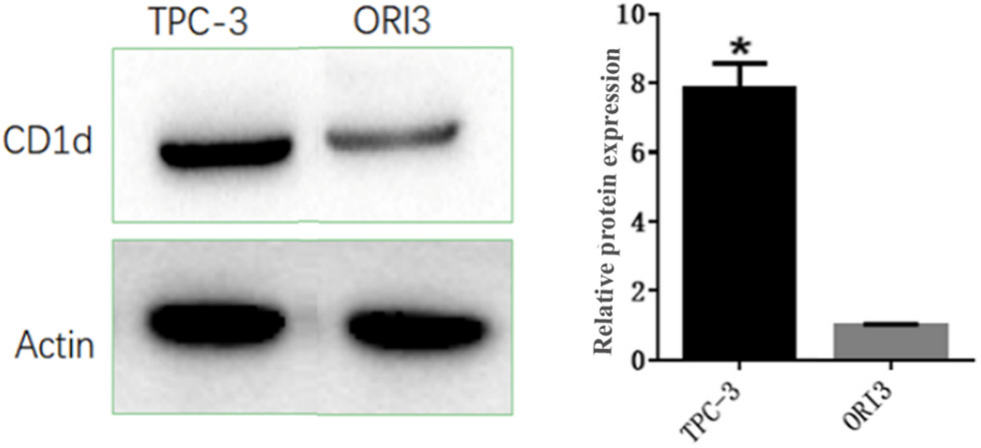
CD1d expression in papillary thyroid carcinoma cells (TPC-1) and human thyroid follicular epithelial cells (Nthy-ori 3-1) detected by western blot analysis. *P < 0.05, compared to Nthy-ori 3-1 cells.

Results from western blot analysis showed that the relative expression of CD1d was significantly higher in the TPC-1 cells than in the Nthy-ori-3-1 cells (*P* < 0.05, Figure 1).

### 3.2 CD1d promotes the proliferation, migration, and invasion of PTC cells

#### 3.2.1 Construction of CD1d-silenced PTC cell lines

**Figure 2:**
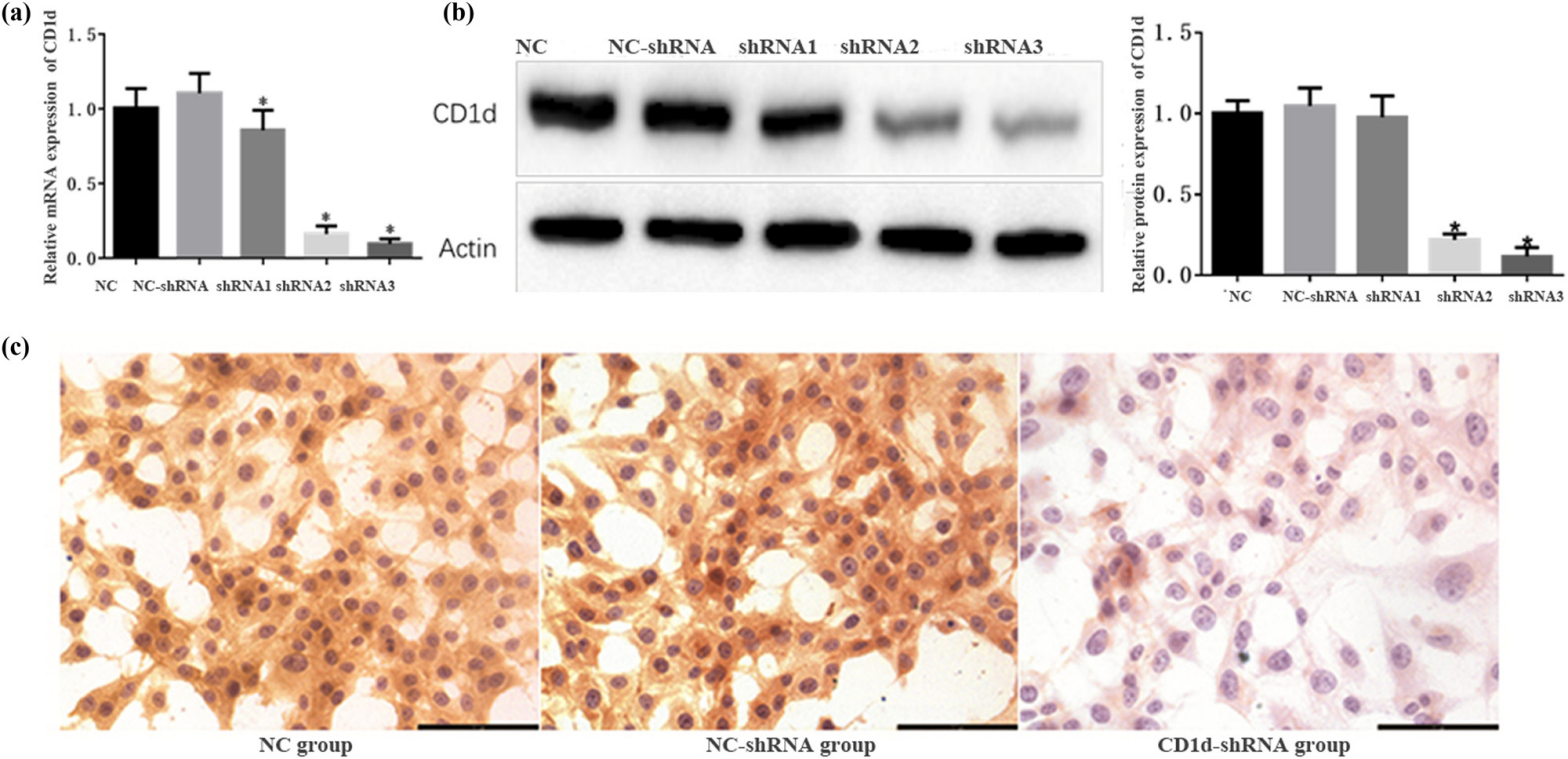
The mRNA and protein expression of CD1d in PTC cells (TPC-1) after transfection with CD1d-shRNA and NC-shRNA detected by (a) qPCR and (b) western blotting analysis. *P < 0.05, compared to the normal control and NC-shRNA groups. (c) CD1d expression in cells of each group detected by immunocytochemical staining (magnification ×400).

After silencing CD1d, the relative expression of CD1d was significantly decreased in the CD1d-siRNA group compared with the NC-siRNA group (*P* < 0.05; Figure 2). The results were further confirmed by the immunocytochemical staining, indicating that CD1d-silenced PTC cell lines were successfully constructed. Cell lines established by transduction of CD1d-shRNA3 and CD1d-shRNA2 vectors were mixed in 1:1 ratio, and were used in the subsequent experiments as CD1d-shRNA group.

### 3.3 Down-regulation of CD1d expression inhibits the proliferation and induces the apoptosis of TPC-1 cells

**Figure 3:**
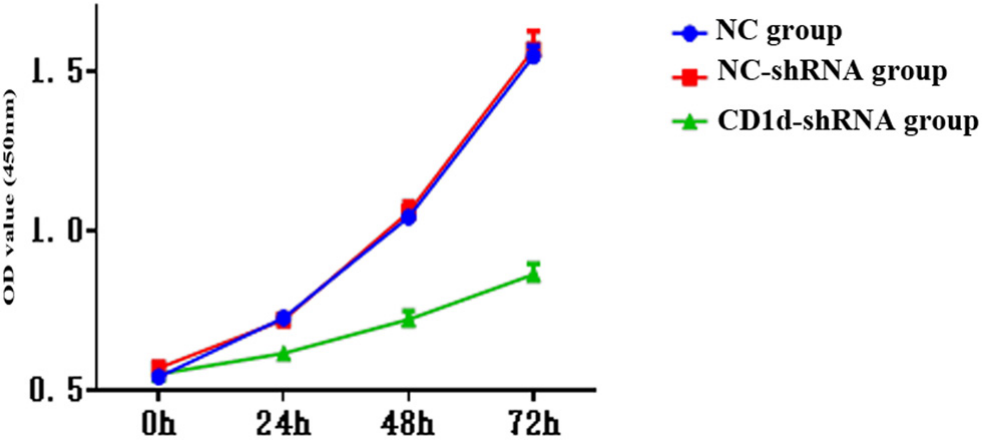
Cell growth curve using CCK8 after down-regulation of CD1d. A significant reduction in cell viability was observed in TCP-1 cells after down-regulation of CD1d expression.

**Figure 4:**
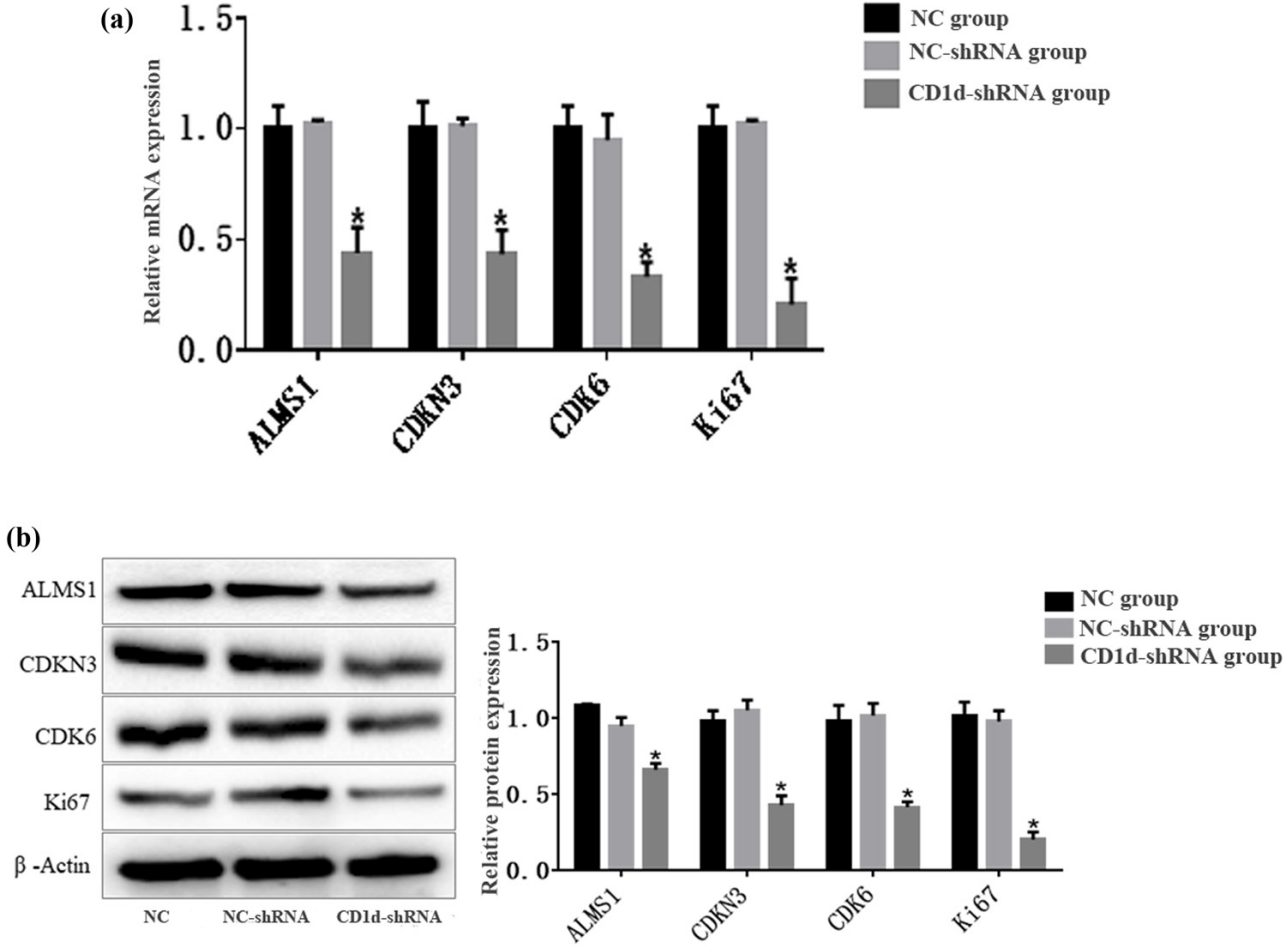
Expression of proliferation-related proteins ALMS1, CDKN3, CDK6, Ki-67 in TCP-1 cells after down-regulation of CD1d. (a) mRNA expression of ALMS1, CDKN3, CDK6, Ki-6. (b) Protein expression of ALMS1, CDKN3, CDK6, Ki-67. *P < 0.05, compared to the normal control and NC-shRNA groups.

**Figure 5:**
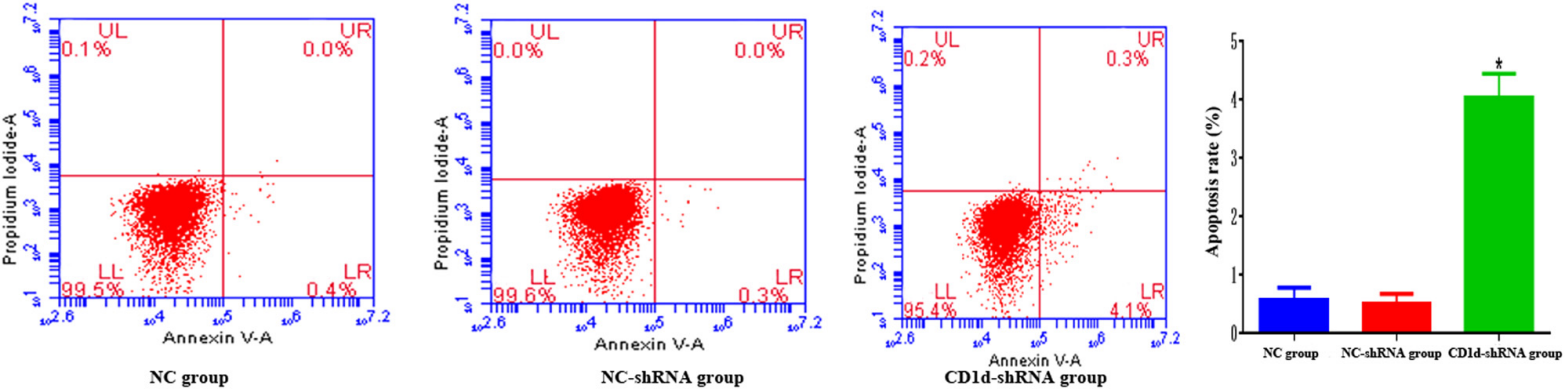
Apoptosis of PTC cells after down-regulation of CD1d. Early apoptosis (Annexin-FITC +/PI−) is shown in the lower right (LR) quadrant, late apoptosis (Annexin V-FITC +/PI +) is shown in the upper right (UR) quadrant, normal cells (Annexin V-FITC-/PI-) are represented in the lower left (LL) quadrant, necrotic cells (Annexin V-FITC-/PI +) are represented in the upper left (UL) quadrant.

**Figure 6:**
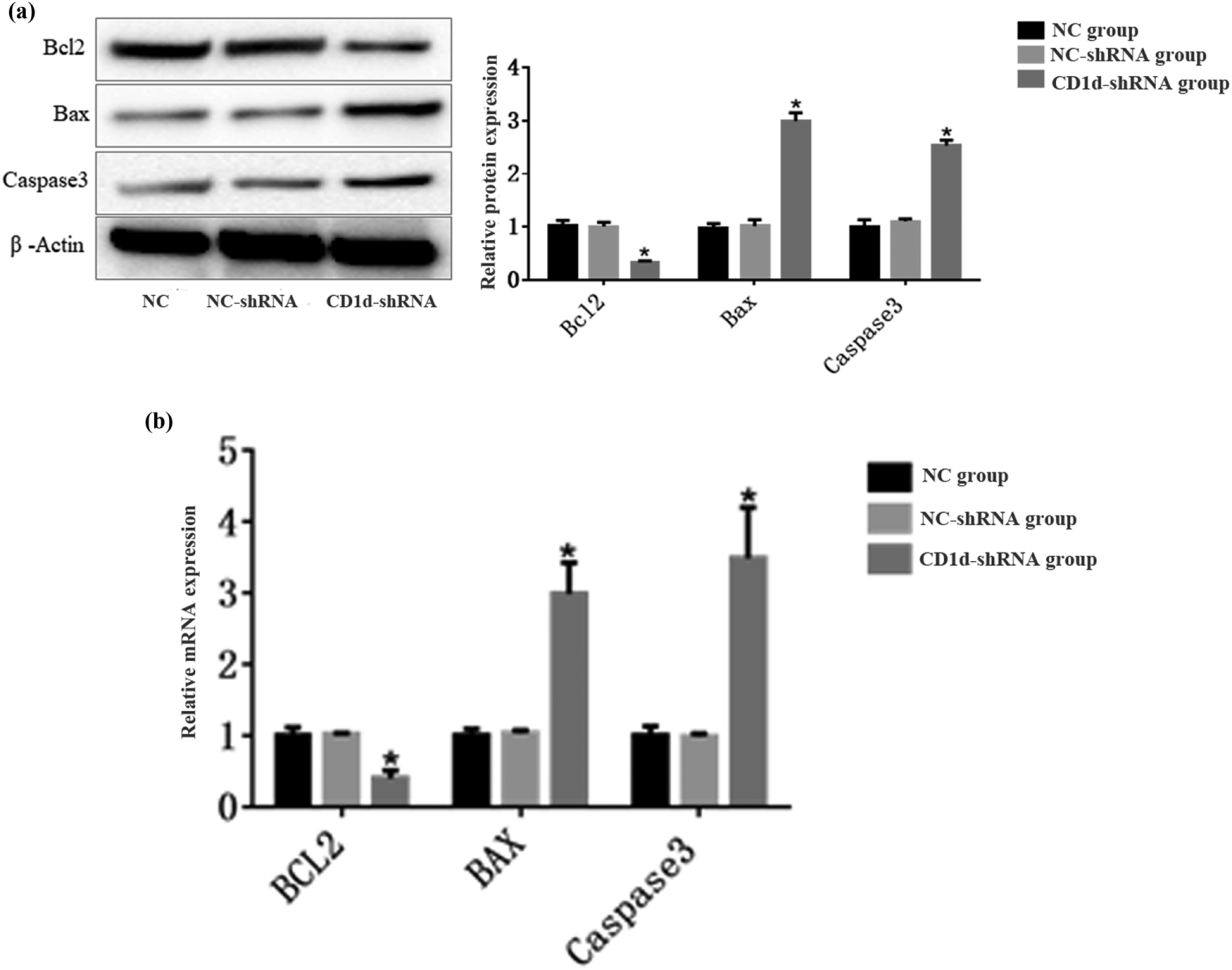
Expression levels of apoptosis-related proteins Bcl2, Bax, and Caspase 3 after down-regulation of CD1d expression. (a) Protein expression of Bcl2, Bax, and Caspase 3. (b) mRNA expression of Bcl2, Bax, and Caspase 3. *P < 0.05, compared to the normal control and NC-shRNA groups.

**Figure 7:**
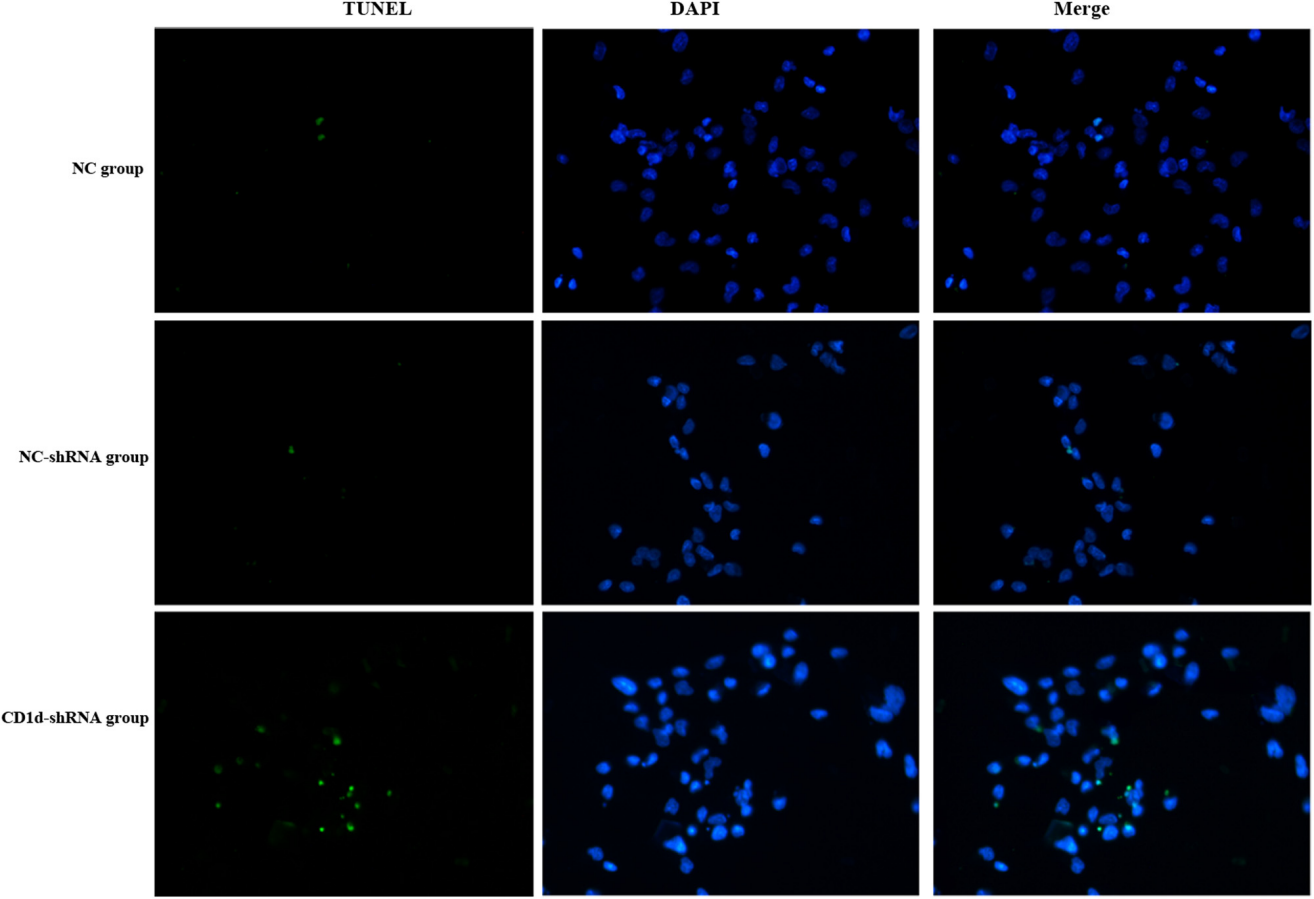
Apoptosis of TCP-1 cells in each group detected by immunofluorescence staining.

CCK-8 results showed that the OD value was significantly lower in the CD1d-shRNA group than those in the normal control and NC-shRNA groups at 48 and 72 h (*P* < 0.05), indicating that down-regulation of CD1d could inhibit the proliferation of PTC cells (Figure 3). To further verify the results, western blot and qPCR analyses were performed to detect the expression of proliferation-associated proteins ALMS1, CDKN3, CDK6, and Ki-67 in cells, the results showed that the expression of proliferation-related proteins was decreased after down-regulation of CD1d (Figure 4), which was consistent with the above-mentioned results. These results indicate that the expression levels of CD1d have an effect on the proliferation of PTC cells. Compared with the NC-shRNA group, the apoptosis rate of PTC cells was significantly increased in the CD1d-shRNA group (*P* < 0.05, Figure 5). To further verify the results, western blot and qPCR analyses were performed to detect the expression of apoptosis-related proteins Bcl2, Bax, and Caspase 3, the results showed that the mRNA and protein expression levels of Bcl2 were significantly decreased, and the mRNA and protein expression levels of Bax and Caspase 3 were significantly increased after down-regulation of CD1d (*P* < 0.05, Figures 6 and 7).

### 3.4 Down-regulation of CD1d inhibits the migration and invasion of PTC cells

**Figure 8:**
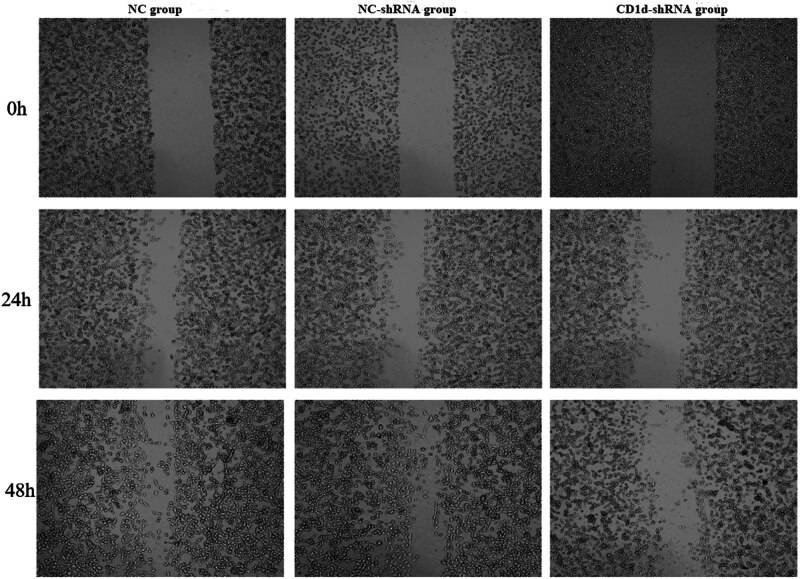
Migration ability of cells in each group detected by scratch assay.

The results from the scratch assay showed that the migration ability of PTC cells was decreased in the CD1d-shRNA group compared with the NC-shRNA group (Figure 8).

### 3.5 GO and KEGG enrichment analysis of DEGs

**Figure 9:**
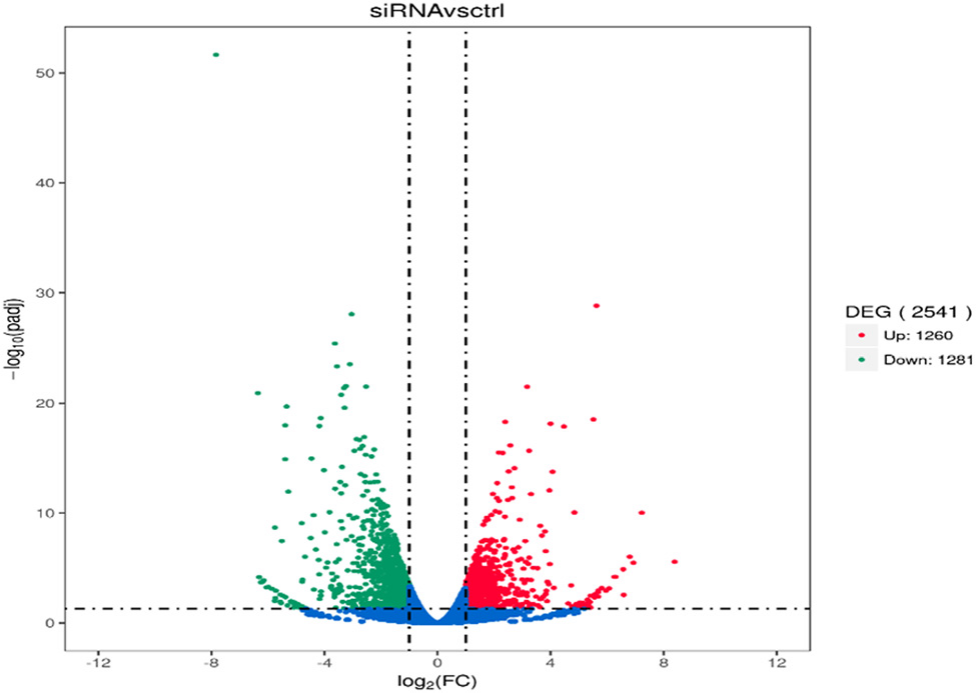
Volcano map of DEGs in the CD1d-siRNA group versus the NC-siRNA group. Red spots represent up-regulated genes, green spots represent down-regulated genes, and blue spots indicate genes with no statistically significant difference.

**Figure 10:**
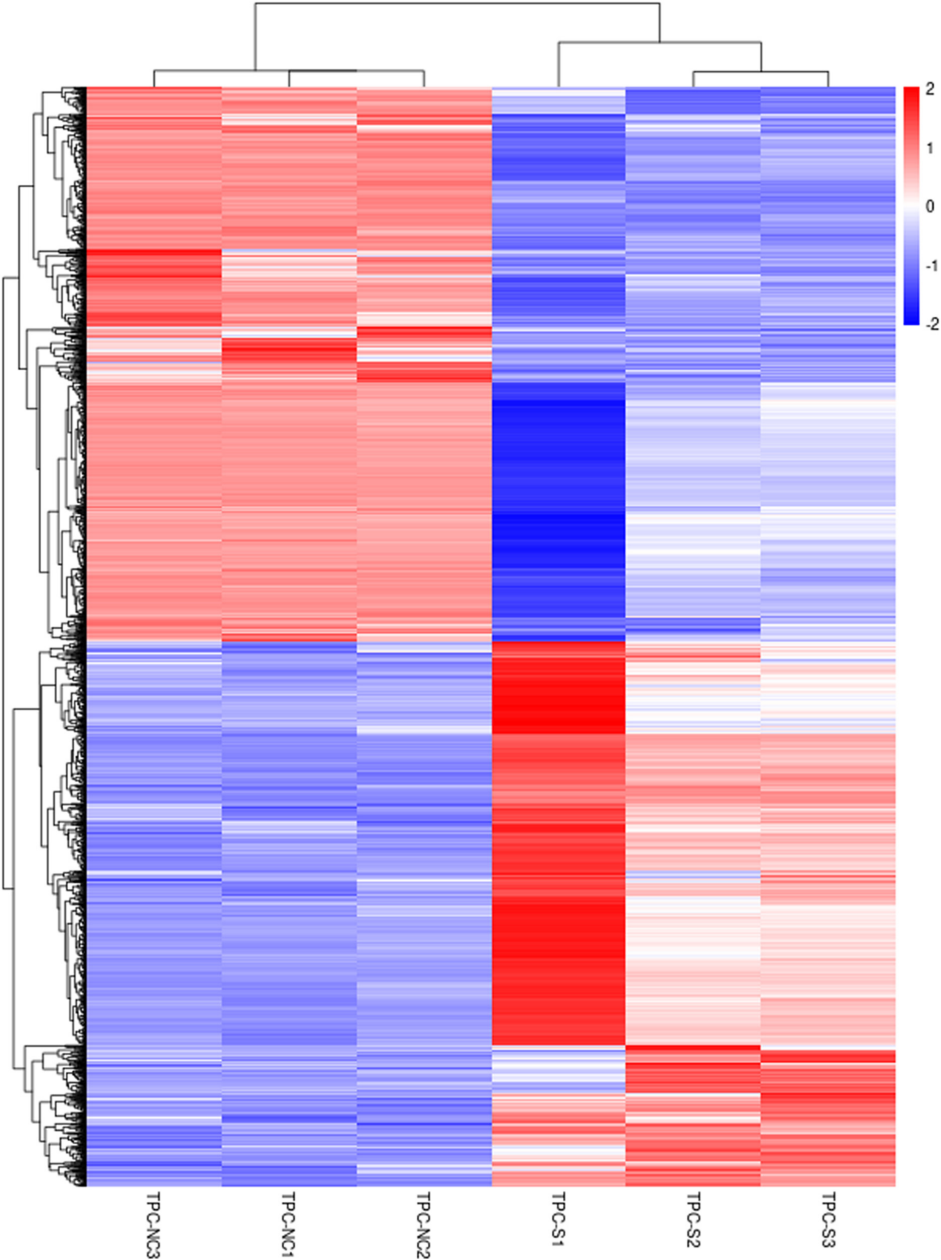
Heat map of DEGs in the CD1d-siRNA group versus the NC-siRNA group. Red represents up-regulated genes and blue represents down-regulated genes.

RNA-seq analysis identified 2,541 DEGs between CD1d-siRNA and NC-siRNA groups, of which 1,260 genes were up-regulated and 1,281 genes down-regulated. The volcano map (Figure 9) and heat map (Figure 10) were drawn to visually show the whole distribution of the DEGs between CD1d-siRNA and NC-siRNA groups.

**Figure 11:**
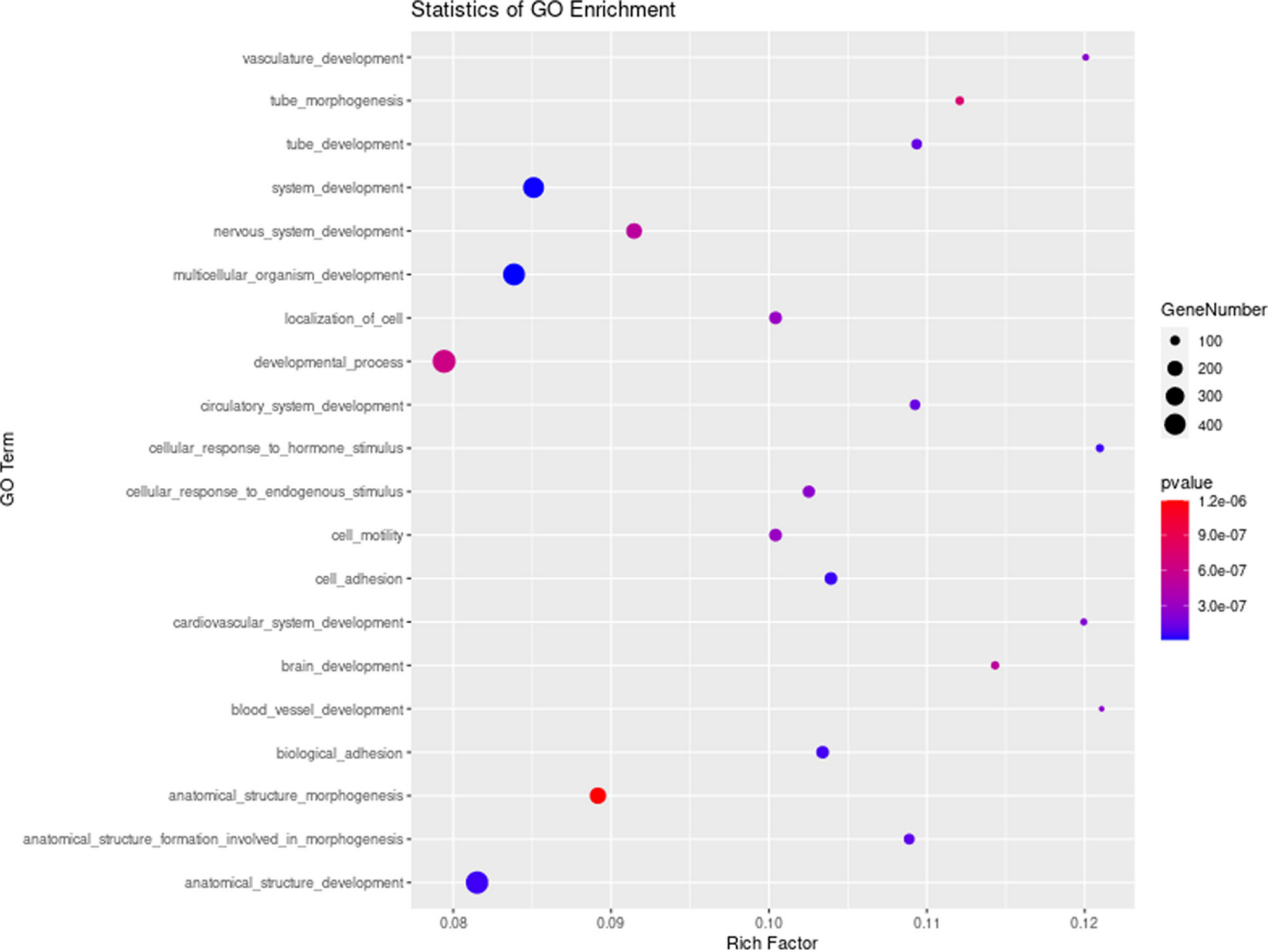
Bubble chart of GO functional annotation analysis of differential expressed genes. The biological processes enriched by differential expressed genes in the CD1d-siRNA group versus the NC-siRNA group.

For GO function annotation analysis, GO terms with *P*-value less than 0.05 were considered significantly enriched by DEGs. The results showed that in biological processes category, 1,017 GO terms were significantly enriched by DEGs, mainly including vascular microenvironment, cell adhesion, apoptosis, and metabolic processes (Figure 11). In the cellular component category, 129 GO terms were significantly enriched, mainly including the plasma membrane and extracellular matrix. And in the molecular function category, 220 GO terms were significantly enriched, mainly including ion binding and transmembrane transport protein.

**Figure 12:**
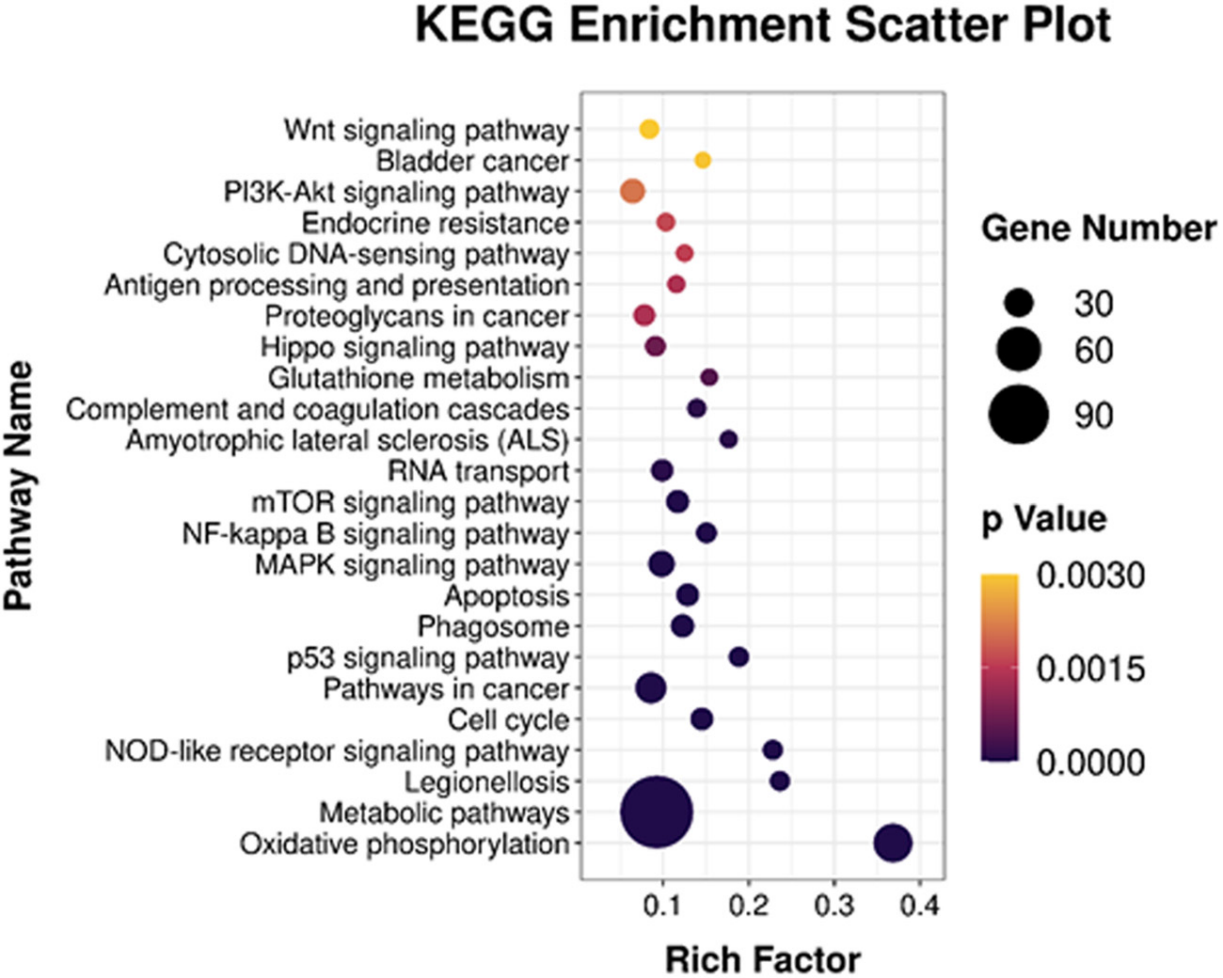
Bubble chart of KEGG pathway enrichment analysis of differential expressed genes between CD1d-siRNA group and NC-siRNA groups.

For KEGG pathway enrichment analysis, KEGG pathways with *P*-values <0.05 were considered as significantly enriched. The results showed that DEGs were mainly enriched in tumor-related signaling pathways, i.e., MAPK and PI3K/AKT signaling pathways (Figure 12).

### 3.6 Down-regulation of CD1d increases the expression of MAPK/NF-κB signaling pathway-related protein in PTC cells

**Figure 13:**
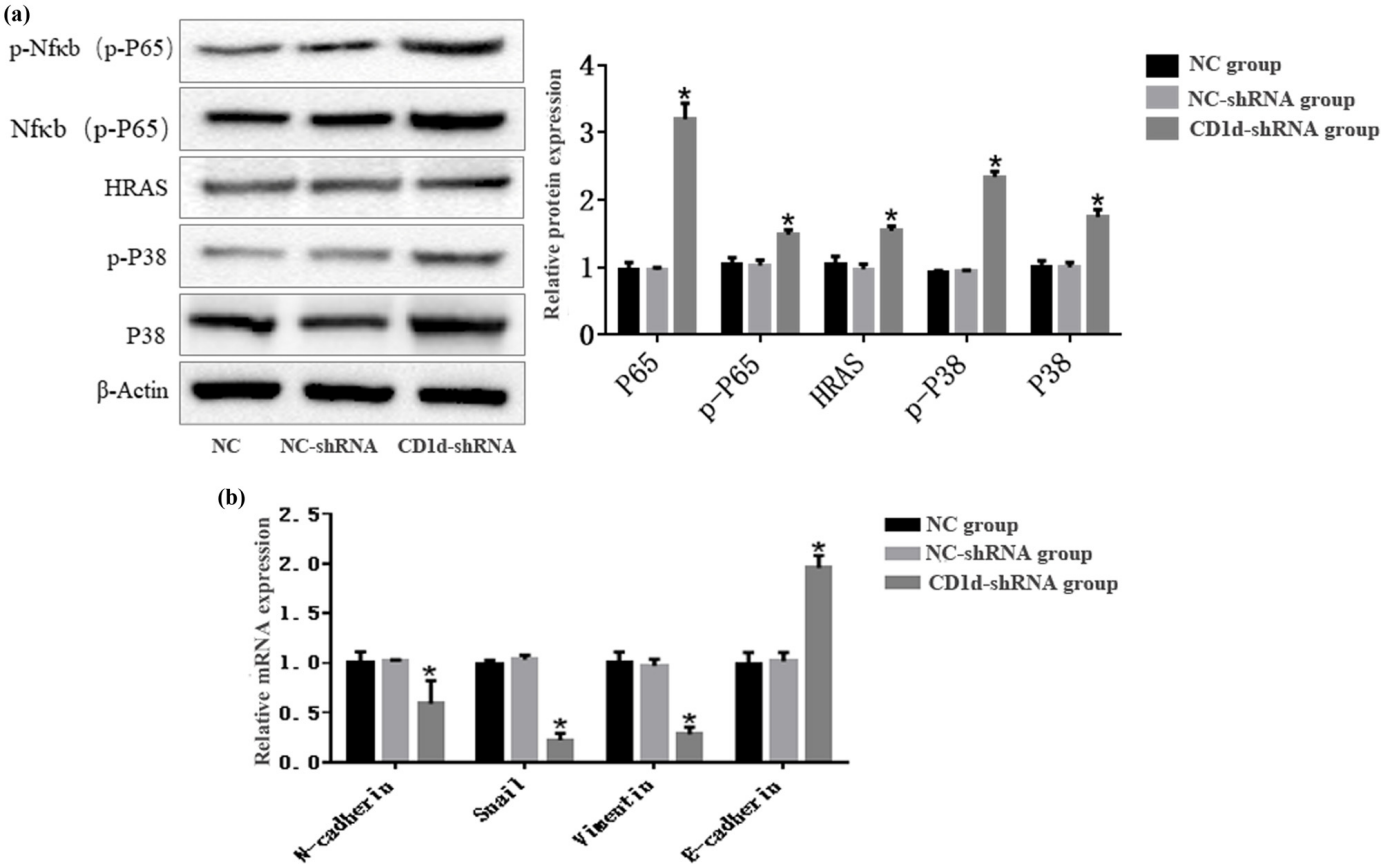
(a) Protein expression of p-NF-κBP65, NF-κBP65, HRAS, p-p38 MAPK, p38 MAPK expression in PTC cells of each group detected by western blot analysis. *P < 0.05, compared to the normal control and NC-shRNA groups. (b) mRNA expression of NF-κBP65, HRAS, p38 MAPK in PTC cells of each group detected by qPCR. *P < 0.05, compared to the normal control and NC-shRNA groups.

Western blot analysis revealed that the protein expression levels of p-NF-κB P65, NF-κB P65, HRAS, p-p38 MAPK, and p38 MAPK in PCT cells were significantly higher in the CD1d-shRNA group compared with the normal control and NC-shRNA groups (both *P* < 0.05, Figure 13a). Results from qPCR showed that the mRNA expression of NF-κB P65, HRAS, and p38 MAPK were significantly higher in the CD1d-siRNA group compared with the normal control and NC-siRNA groups (both *P* < 0.05, Figure 13b).

## 4 Discussion

There have been some advances in the application of targeted therapeutic agents for thyroid cancer, which provide clinical benefits. The occurrence and development of PTC are complex processes involving multiple genes and cellular pathways. Alterations in expression stability of genes can cause abnormalities in different signaling pathways, such alterations could promote tumorigenesis and development of several types of cancer, alter the migration and proliferation abilities of cancer cells, change the process of apoptosis and cell cycle, thus ultimately leading to cancer progression. For changes in genes in PTC, multiple genes were often involved rather than a single gene. Genes that are differentially expressed are interacted with each other to form a network, which play a key role in the occurrence and development of PTC.

Recently, CD1d has been found to be aberrantly expressed in several types of cancer, and plays an important role in cancer progression, demonstrating its potential as a new molecular biomarker for tumor diagnosis, prognosis, and treatment. PTC accounts for 90% of all thyroid cancers with complex genomic heterogeneity. Although sorafenib is FDA approved for the treatment of PTC, a randomized phase III trial of sorafenib in patients with thyroid cancer posed concern about the occurrence of adverse events, this may cause an obvious reduction in patients’ quality of life [15]. There is also an urgent need for targeted cancer drugs and identification of cancer driver genes.

The results of the present study showed that CD1d expression was significantly elevated in PTC cells. The effect of CD1d on PTC cells is unclear. An increasing number of studies have reported that CD1d could act as tumor suppressor gene or pro-oncogenic gene, and exerts regulatory effect on cell function and gene expression through various mechanisms [16]. Aberrant methylation of CD1d gene in gastric cancer tissues of patients with gastric cancer is considered to be significantly correlated with the clinical staging of gastric cancer. The aberrant methylation of CD1d gene was detected in 35% of pathological tissue specimens obtained from patients with atrophic gastritis, suggesting that the elevated rate of aberrant methylation of CD1d gene in patients with atrophic gastritis may serve as an early warning sign for gastric cancer, which is beneficial for early prevention and treatment of gastric cancer. In a previous study of renal cell carcinoma [17], gene expression microarray showed significant expression of CD1d in renal cell carcinoma compared with renal tissues, immunocytochemical staining showed that CD1d expression was significantly associated with tumor stage/grade, higher recurrence rates, poorer cancer-specific, and overall survival, suggesting that CD1d expression is strongly correlated with clinical outcomes. The present study investigated the role of CD1d in PTC cells, the results showed that down-regulation of CD1d expression significantly reduced the proliferation, migration, and invasion abilities of PTC cells, and induced cell apoptosis. These results indicate that CD1d acts as a pro-oncogenic gene in PTC cells, and plays a regulatory role in the progression of PTC cells.

MAPK pathway has been shown to play an important role in various cellular processes, such as cell growth, development, and response to changes in the intracellular environment. NF-kB family is closely related to tumor development, which is aberrantly expressed and activated in various types of cancer cells. Aberrant NF-κB expression in non-small cell lung cancer, nasopharyngeal cancer, esophageal cancer, cervical cancer, and colorectal cancer has been reported [18–22]. The relationship and interaction between MAPK and NF-κB signaling pathways have also been reported. MAPK, also known as extracellular signal-regulated kinase (ERK), can activate NF-κB by regulating ERK phosphorylation cascade, thus regulating the transcription and expression of related downstream factors [23–25]. Additionally, MAPK and NF-κB signaling pathways may play an important role in regulating the inflammatory response in microvascular endothelial cells, a response that may be associated with the inflammatory response caused by tumor resistance to chemotherapy and radiotherapy [26–28].

In this study, KEGG pathway enrichment analysis of DEGs showed that CD1d could affect tumor development via regulating the MAPK and NF-κB signaling pathways. Further experiments revealed the involvement of the MAPK and NF-κB signaling pathways in the regulation of proliferation and differentiation of TCP-1 cells after down-regulation of CD1d. Results from western blot analysis showed that the protein expression levels of p-NF-κB p65, NF-κB p65, p-p38 MAPK, p38 MAPK, and HRAS were significantly elevated in the CD1d-shRNA group compared with normal control and NC-shRNA groups. HRAS is one of the factors stimulating MAPK signaling pathway activation, the elevated expression of HRAS suggests that CD1d may contribute to tumor growth by activating phosphorylation levels of the above-mentioned MAPK/NF-κB signaling pathway-related proteins. The MAPK/NF-κB pathway is considered to be closely related to tumorigenesis and development. Through analysis of transcriptome results and verification of related pathway proteins in this study, we speculated that CD1d may cause the progression of thyroid papillary carcinoma through the MAPK/NF-κB pathway.

However, this study still has limitations. This is an *in vitro* study using two cell lines. The use of cell lines often cannot mimic the complex situations of patients well, thus limiting the translational potential of such type of study. The findings observed in this study need to be further validated in *in vivo* animal experiments in the future.

In conclusion, our findings suggest that CD1d can affect the proliferation, migration, and apoptosis of PTC cells possibly via the MAPK/NF-κB signaling pathway, which might serve as a new molecular target for the treatment of PTC. Further studies are needed to explore whether CD1d gene can affect the proliferation, apoptosis, and autophagy of cancer cells through regulating other pathways.
